# Genetic and Biological Effects of ICAM-1 E469K Polymorphism in Diabetic Kidney Disease

**DOI:** 10.1155/2020/8305460

**Published:** 2020-06-12

**Authors:** Xiuli Zhang, Norhashimah Abu Seman, Henrik Falhammar, Kerstin Brismar, Harvest F. Gu

**Affiliations:** ^1^Department of Nephrology, The second People's Hospital, Shenzhen, The first Affiliated Hospital of Shenzhen University, Guangdong 518000, China; ^2^Cardiovascular, Diabetes and Nutrition Research Center, Institute for Medical Research, Kuala Lumpur 50588, Malaysia; ^3^Rolf Luft Research Center for Diabetes and Endocrinology, Department of Molecular Medicine and Surgery, Karolinska Institutet, Stockholm 17176, Sweden; ^4^Department of Endocrinology, Metabolism and Diabetes, Karolinska University Hospital, Stockholm 17176, Sweden; ^5^Center for Pathophysiology, School of Basic Medicine and Clinical Pharmacy, China Pharmaceutical University, Nanjing 210009, China

## Abstract

Diabetic kidney disease (DKD) is a complex disease, in which local inflammatory stress results from both metabolic and hemodynamic derangements. Intercellular adhesion molecule 1 (ICAM-1) is an acute-phase protein marker of inflammation. In the recent years, clinical observations have reported that increased serum/plasma ICAM-1 levels are positively correlated with albuminuria in the patients with type 1 (T1D) and type 2 diabetes (T2D). Genetic association studies have demonstrated that genetic polymorphisms, including SNP rs5498 (E469K, G/A), in the *ICAM1* gene is associated with DKD. rs5498 is a nonsynonymous SNP and caused by substitution between E (Glu) and K (Lys) for ICAM-1 protein. In this review, we first summarized the genetic effects of *ICAM1* E469K polymorphism in DKD and then demonstrated the possible changes of ICAM-1 protein crystal structures according to the genotypes of this polymorphism. Finally, we discussed the genetic effects of the *ICAM1* E469K polymorphism and the biological role of increased circulating ICAM-1 protein and its formation changes in DKD.

## 1. Introduction

Diabetes has become a global epidemic and a large proportion of diabetes patients develop microvascular complications. Diabetic kidney disease (DKD) is a prevalent microvascular complication and presents in approximately 25–40% of patients with long-standing diabetes [1, 2]. This complication is characterized with progressive proteinuria from microalbuminuria to persistent proteinuria and confers additional risk of cardiovascular disease and mortality. DKD is the leading global cause of end-stage renal disease, a condition which requires treatment with dialysis or kidney transplantation [2, 3]. The treatment costs of patients with DKD are increasing and impose a substantial burden on the healthcare system. However, as is the case with the majority of complex diseases, identifying causal genetic variants contributing to DKD has proven difficult. A better understanding of the heritable genetic factors underlying DKD by using not only genetic studies but also biological functional analysis may lead to the discovery of new biomarkers of disease susceptibility and novel therapeutic strategies.

Although the pathogenesis in DKD is multifactorial, local inflammatory stress results from both metabolic and hemodynamic derangements [4, 5]. Intercellular adhesion molecule 1 (ICAM-1, OMIM: 147840) is a 90 kD acute-phase protein marker of inflammation. It is an inducible surface glycoprotein expressed in leukocytes, endothelial, and other cell types that promotes adhesion in immunological and inflammatory reactions [4–6]. To better understand the genetic and biological effects of ICAM-1 in DKD, we have performed the genetic studies of the ICAM1 gene in Swedish patients with type 1 diabetes (T1D) and also in Malays type 2 diabetes (T2D) with or without DKD [7, 8]. According to the genotypes of *ICAM1* genetic polymorphisms, we have analyzed the crystal structures of ICAM-1 protein by using the molecular graphics program PyMol [9–11]. In this article, we focused on the evaluation of the genetic effects of the *ICAM1* E469K polymorphism in DKD and also demonstrated the possible biological changes of ICAM-1 protein crystal structure according to the genotypes of this polymorphism. Based upon that, we summarized the genetic and biological effects of the *ICAM1* E469K polymorphism in DKD, which may provide useful information for better understanding the pathophysiology of the disease.

### 1.1. The *ICAM1* Gene and Its E469K Polymorphism in DKD

The *ICAM1* gene locates to chromosome 19p13.2 in human. Genetic linkage analyses in T1D, T2D, and DKD have indicated that this chromosomal segment resides in a region linked to diabetes. Based upon a genome-wide scan study in 93 affected sib-pairs and 263 multiplex families from the UK, Mein et al. reported that a chromosomal region of 19p13 was linked to T1D [12]. Later on, this linkage with T1D in chromosome 19p13 was replicated by the study of 2658 affected sib-pairs in the USA [13]. The chromosomal region of 19p13 was also found to be linked with several lipid-related traits, including total cholesterol, triglycerides, and low-density lipoprotein in T2D according to the studies of Caucasian, African-American, and Hispanic families [14–16]. All these studies demonstrated that the loci in chromosome 19p13 might confer the susceptibility to diabetes and diabetic vascular complications. Interestingly, there are several genes, including *INSR* (Insulin receptor), RETN (Resistin), and *ICAM1*, located in this chromosomal region.

**Figure 1:**
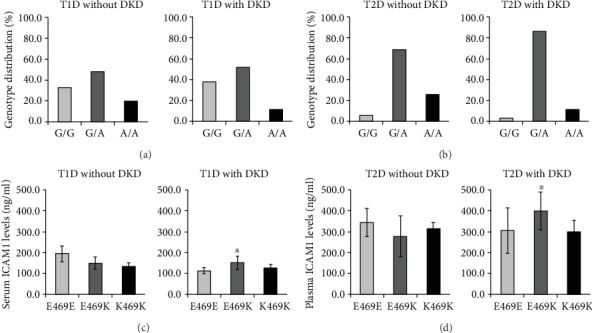
Genotype distribution of the *ICAM1* E469K polymorphism and circulating ICAM-1 levels in type 1 and type 2 diabetes with or without diabetic kidney disease. Genotype distribution patterns (top) showed a high frequency of heterozygous genotypes in type 1 diabetes (T1D) subjects with or without diabetic kidney disease (DKD) (Figure 1(a)) and type 2 diabetes (T2D) patients with or without DKD (Figure 1(b)). According to the genotypes, serum/plasma ICAM-1 levels were analyzed (bottom). In T1D subjects with DKD, the heterozygous *ICAM1* E469K carriers had higher circulating ICAM-1 levels than what in homozygous E469E and K469K carriers (*P* < 0.05, adjusted for age and sex) (Figure 1(c)). The similar finding was found in T2D patients with DKD (*P* < 0.05, adjusted for age and sex) (Figure 1(d)). Data were replicated and modified from Ma et al. 2006 and Abu Seman et al. 2013.

In 2006, we performed a genetic association study of the *ICAM1 g*ene in Swedish subjects with normal glucose tolerance (NGT), T1D with, or without DKD. Six single nucleotide polymorphisms (SNPs), including three Tag SNPs rs5498 (E469K A/G), rs1799969 (R241G, A/G), and rs281432 (C/G), in the *ICAM1* gene were selected for genotyping experiments based upon the information of their position and function in dbSNP. The SNP rs5498 is a nonsynonymous SNP and caused by substitution between GAG for E (Glu) and AAG for K (Lys) in the 469^th^ codon of ICAM-1 protein (position 10285007 of chromosome 19, contig ID NT_011295.12, from the version of GRCCh38.p7). The *ICAM1* gene consists of 7 exons and the E469K polymorphism is located in the 6^th^ one [6, 17]. There is high linkage disequilibrium (LD) in the gene region covering exons 5 and 6 but LD from promoter to intron 4 is low. The 7^th^ exon has a large 3′-UTR. Genotyping experiments were conducted with a high-throughput SNP scoring technique called dynamic allele-specific hybridization (DASH) [18], which was the first genotyping protocol that we have used in our laboratory. Results showed that there was a significant association of SNP rs5498 (E469K) in the *ICAM1* gene between the subjects with NGT and T1D patients in Swedish population. Further analysis between T1D with and without DKD indicated that this polymorphism was associated with DKD. Interestingly, we found a high heterozygous index of this polymorphism presenting in this population [7]. About 10 years later, we replicated the genetic association study of the *ICAM1* gene in Malays T2D subjects with or without DKD, and the genotyping experiments were done by using TaqMan allelic discrimination. The high heterozygous index of ICAM1 E469K polymorphism was also found in Malays population [8]. With the extension of sample collection and follow-up study, our studied cohorts were enlarged. Thereby, we were able to further evaluate the genotyping data by using TaqMan allelic discrimination. Therefore, Figure 1 in this paper was modified from our previous publications [7, 8] and represented the genotype distribution of the *ICAM1* E469K polymorphism, respectively, in Swedish T1D (Figure 1(a)) and Malays T2D subjects with or without DKD (Figure 1(b)). As seen in the figure, the frequency of heterozygous E469K genotype was higher than the frequencies of both homozygous genotypes, i.e., E469 and 469K.

Once the high heterozygous index in genotyping distribution was seen, several questions had been taken into our consideration. First, was the high heterozygous index of the *ICAM1* K469E polymorphism caused by genotyping error? We replicated genotyping experiments for all Swedish subjects but using another technique named pyrosequencing [19], and the genotyping results were fully matched. Thereby, the possibility of a high heterozygous index caused by genotyping error was excluded. Second, was the high heterozygous index of the *ICAM1* K469E polymorphism induced by genomic duplicons in the *ICAM1* gene? Several reports of human genome analyses have demonstrated that segmental duplications (duplicons) with >90% similarity between two copies may comprise at least 5% of g.DNA in human [20–22]. If a SNP is involved into the region of duplicons, a high heterozygous index may be presented in the genotype distribution. We conducted sequencing analyses along the *ICAM1* gene in the studied subjects, and no duplicated sequence in the gene region was found. Third, did the high heterozygous index of the *ICAM1* E469K polymorphism present in other populations? According to the record in HapMap, the highest genotype frequency of the *ICAM1* E469K polymorphism in 226 European Caucasian individuals was heterozygous at 46.0% [23], which was similar with the genotyping data in Swedish population, while the heterozygous frequency of the *ICAM1* K469E polymorphism in Malays population was even higher (Figures 1(a) and 1(b)). Before we reported the high heterozygous index of *ICAM1* E469K polymorphism [7], there were several genetic association studies of *ICAM1* E469K polymorphism in Danish, Finnish, and Japanese T1D subjects [24–26]. However, the information concerning the genotype distribution of this SNP in Danish and Finnish population was unclear. By the personal communications, we found that the phenomenon of high heterozygous index of this SNP exists in all studied populations.

### 1.2. Circulating ICAM-1 and Its Formation Changes according to the E469K Polymorphism

DKD is a progressive disease, in T1D and T2D patients as a result of increased urinary albumin excretion (UAE) rate and compromised renal function. The early phase of microalbuminuria could be reversed, while the reduction of renal function begins with proteinuria. Clinical observation has demonstrated that high circulating ICAM1 levels are associated with DKD, and all caused mortality and cardiovascular morbidity in T1D patients [27, 28]. Similarly, serum/plasma ICAM-1 levels were found to be correlated with albuminuria in T2D patients [29–31]. At the same time, experimental studies with diabetic animal models have supported these clinical observations and demonstrated that serum ICAM-1 levels in streptozotocin-induced rats were increased in parallel with the elevation of UAE [32]. Furthermore, ICAM-1 was found to be overexpressed in tubular epithelial cells of kidney in T2D db/db mice and in glomeruli of diabetic rats [33, 34].

**Figure 2:**
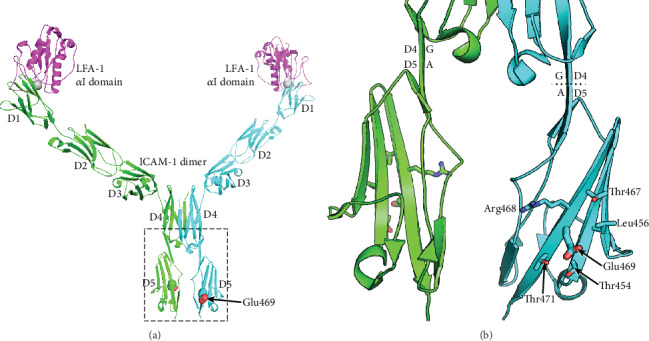
Structural context of the *ICAM1* E469K polymorphism. The structures are shown as ribbon cartoon diagrams. (a) Model of the dimeric ICAM-1 ectodomain bound to the *α*I domain of integrin LFA-1 reconstructed by combining two crystal structures. Glu-469 in domain 5 is shown with its sidechain atoms as spheres. The Mg^2+^ ion at the center of the LFA-1/ICAM-1 binding site is shown as a silver sphere. (b) Detailed view of the region boxed in (a). Sidechains of Glu-469 and nearby residues are shown in stick representation, and Glu-469 is emphasized with thicker stick. The boundary between the G *β*-strand in D4 that continues into the A *β*-strand in D5 is shown with a dotted line in the cyan ICAM-1 monomer.

ICAM-1 proteins act as ligands and the primary receptors for ICAM-1 are integrins for mediating cell–cell interactions and signal transduction. However, ICAM-1, unlike most integrin-binding proteins, does not contain an RGD (Arg-Gly-Asp) motif to promote integrin binding but is targeted to two integrins of the *β*2 subunit family, i.e., leukocyte adhesion protein-1 (LFA-1) and Mac-1 (integrin, alpha M) [35, 36]. Thus, ICAM-1 has a role not only for T lymphocytes activation but also for leukocyte–endothelial cell interaction. Considering the presentation of high heterozygous index of *ICAM1* E469K polymorphism, we have for several years paid the attention for analysis of biochemical structure and functional changes of ICAM-1 protein according the genotypes of this polymorphism. We thus used the molecular graphics program PyMol to analyze the formation of ICAM-1 protein. ICAM-1 is composed of five immunoglobulin-like domains (D1-D5), a transmembrane domain with a Gly-X-X-Gly dimerization motif, and a cytoplasmic domain that binds ezrin-radixin-moesin family adaptors that link to the actin cytoskeleton [37–40]. D1 and D3 of ICAM-1 bind to the integrins LFA-1 and Mac-1, respectively. A substantial portion of ICAM-1 on the cell surface dimerizes by fusion of each of the two *β*-sheets in D4 into a super *β*-sheet (Yang et al. 2004). The structure of a domain 3-domain 5 (D3-D5) fragment of ICAM-1 has been revealed in both monomeric and dimeric forms (Figure 2(a)). The structure of a D1-D2 fragment of ICAM-1 reveals how the *α*I domain of integrin LFA-1 binds to D1 [41]. At the center of the interface, a Mg^2+^ ion held by the *α*I domain binds to an acidic residue, Glu-34, in an edge *β*-strand of D1 of ICAM-1. Crystal structures of ICAM-1 containing domains 3, 4, and 5 [42] (Protein Databank ID 1p53 and 2 oz4) were visualized. Residue numbering in these structures is according to the mature sequence after removal of a 27-residue signal peptide; numbering in Figures 2(a) and 2(b) follows the immature sequence consistent with the nomenclature used in genetics. The E469K polymorphism locates to domain 5 of ICAM-1. Furthermore, the E-469 sidechain in the D4-D5 crystal structure protrudes from a face of D5 that is rotated about 90° away from the dimer interface. In the K-469 polymorphism, the K sidechain will protrude in a similar direction. Sidechains in the vicinity of residue 469 are shown in Figure 2(b).

### 1.3. Prediction of Genetic and Biological Effects of the E469K Polymorphism in DKD

Ours and other groups have found that the *ICAM1* E469K polymorphism represents a high heterozygous frequency in the genotype distribution of the studied populations [7, 8, 24–26]. This high heterozygous frequency in genotype distribution is unlikely an error due to genotyping limitation or genomic duplication. We have demonstrated that DKD subjects with heterozygous genotype have increased circulating ICAM-1 levels, indicating that the *ICAM1* E469K polymorphism heterozygous is most likely involved in the pathogenesis of DKD.

To understand whether the *ICAM1* E469K polymorphism heterozygous has a biochemical functional role in ICAM-1 protein, we have carried out a structural analysis.  Figure 2 shows a model of the dimeric form of ICAM-1 bound to LFA-1 *α*I domains, constructed from separate crystal structures of LFA-1 *α*I complexed to D1-D2 and the D3-D5 dimer. The E469K (immature sequence numbering) polymorphism locates to D5 of ICAM-1. Glu-469 is well exposed to solvent in a central location in *β*-strand G in D5. *β*-strand G is present in one of the two *β*-sheets of D5, which contains from one edge to the other the A′, G, F, C, and C′ strands [40]. *β*-strand G is the last segment of D5 and is followed by a linker of 4 residues and the TM domain. Because of the dyad symmetry of the ICAM-1 dimer, its average orientation on the cell surface will be with the long axes of D4 and D5 normal to the membrane bilayer. This orientation of ICAM-1 dimers is shown in Figure 2; in this view, the membrane would locate horizontally and perpendicular to the plane of the page. The orientation between D4 and D5 is maintained by a continuous *β*-strand formed by *β*-strand G in D4 and A in D5. Thus, although in the ICAM-1 dimer the D4-D4 interface is extensive and the D5-D5 interface is small, the stable orientation between D4 and D5 provides confidence that the monomer-monomer interface at D5 of ICAM-1 dimers on cell surfaces. There are no significant interactions between any of the neighboring residues that would be perturbed by the polymorphism. The E469K polymorphism is therefore expected to have no effect on the equilibrium between monomeric and dimeric forms of ICAM-1 on the cell surface. The polymorphism also locates distal from the integrin-binding sites in D1 and D3 and might disrupt the protein biochemical function.

We speculate that it may affect the formation of higher-order assemblies of ICAM-1 on the cell surface. Interaction between cells bearing LFA-1 and ICAM-1 results in the clustering of ICAM-1 at sites of cell-cell adhesion. Clustering has been visualized both at immunological synapses and in “cups” that form around lymphocytes that are migrating through endothelial cells [39]. Clustering is likely to be enhanced by association of integrins with the actin cytoskeleton through adaptors such as talin and kindlin and association of ICAM-1 with the actin cytoskeleton through ezrin-radixin-moesin family members. “Strings” of ICAM-1 may form on the cell surface through dimerization of ICAM-1 both at the D4-D4 interface and a weaker D1-D1 interface [40]. We speculate that ICAM-1 strings laterally associate to form higher-order assemblies through an interface in which Glu-469 participates. Glu-469 protrudes from a relatively flat surface on D5 that bears three Thr residues of modest hydrophobicity and the hydrophobic residue Leu-456 (Figure 2(b)) that make this face of D5 a candidate for an interface in higher-order lateral association of strings. Because Lys is opposite in charge from Glu, the polymorphism might disrupt such a lateral association. Glu-469 has no specific interactions such as hydrogen bonds with neighboring residues, so an effect on the stability of D5 or ICAM-1 of the polymorphism seems unlikely. We speculate that Glu-469 is involved in the formation of higher-order ICAM-1 assemblies on cell surfaces, an idea that requires further investigation. The question why DKD subjects heterozygous have increased circulating ICAM-1 levels still remains; although, we have demonstrated the *ICAM1* E469K polymorphism has the effects in structure and biochemical functional changes of ICAM-1. It may be necessary to purify the ICAM-1 protein from serum or plasma samples of DKD subjects heterozygous for further 3D protein structural and functional analyses.

There are several reports concerning the heterozygous carriers with increased phenotypes. Previously, Leiber et al. have demonstrated that the 50% of the heterozygous carriers of MEFV mutations have increased serum concentrations of neutrophil-derived protein S100A12 [43]. Recently, Taillandier et al. have reported that the Pro549Ala heterozygous carriers of COL1A2 have more serious phenotypes of hypophosphatasia, mainly due to the dominant-negative effects [44]. Zhu et al. have identified a novel heterozygous variant, which leads to the formation of a truncated COL4A4 protein in Alport Syndrome [45]. Moreover, Serra-Juhe et al. have showed that heterozygous rare variants in several candidate genes of the melanocortin pathway have strong effects in nonsyndromic severe obesity [46]. Therefore, the genetic effects of heterozygous of *ICAM1* E469K and its related functional changes that we have been reported in the present study may not be the findings “by chance.” Liu et al. have conducted a meta-analysis for *ICAM1* E469K polymorphism and DKD in several cohorts by using allelic, dominant, recessive, and additive models. The statistically significant heterogeneity (I^2^ ranged from 62-95) across the studies is observed [47].

**Figure 3:**
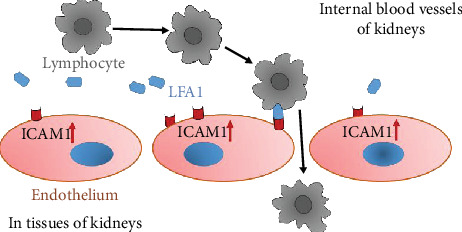
Possible cellular mechanism of ICAM1 in the development of diabetic kidney disease. This schematic diagram implicates that *ICAM1* DNA transcription in the nuclei and mRNA translation in plasma of endothelium cells is increased under a diabetic condition with hyperglycemia. Subsequently, the ICAM-1 protein expression on the surface of endothelium cells is upregulated. ICAM-1 protein binding activity with leukocyte adhesion protein-1 (LEA-1), which is from blood, is increased, while heterozygous of ICAM-1 E469K protein likely more actively binds with LFA-1. Thereby, more and more lymphocytes from blood due to combining ICAM-1 and IFA-1 are transferred through endothelium cells in glomeruli and peritubular capillaries of the nephron in the kidney. Consequently, injure of kidney glomeruli and tubular has occurred.

Evidence from experimental studies with animal models for diabetes has indicated that ICAM-1 is overexpressed not only in glomeruli diabetic rats (48) but also in tubular epithelial cells of kidney in T2D db/db mice (49).  Figure 3 is a schematic diagram to implicate the possible cellular mechanism of ICAM-1 in the development of DKD. In a diabetic condition with hyperglycemia, the *ICAM1* DNA transcription in the nuclei and mRNA translation in plasma of endothelium cells are increased. Subsequently, the ICAM-1 protein expression on the surface of endothelium cells is upregulated. There are leukocyte adhesion protein-1 (LEA-1) in blood. ICAM-1 protein binding activity with LFA-1 is increased, while heterozygous of ICAM-1 E469K protein possibly more actively binds with LFA-1. Thereby, more and more lymphocytes from blood due to combining ICAM-1 and IFA-1 are transferred through endothelium cells in glomeruli and peritubular capillaries of the nephron in the kidney. Consequently, injure of kidney glomeruli and tubular has occurred, and the proteins are excreted to urine.

### 1.4. Summary and Perspective

Taking together the evidence from genetic studies and biochemical functional analyses, we propose that the heterozygous carriers of the *ICAM1* E469K polymorphism among the patients with DKD have increased circulating ICAM-1 protein, while the formation of ICAM-1 protein and related biological function are changed. ICAM-1 plays a pathophysiological role in the development of DKD as previously described [6]. Under a diabetic condition with hyperglycemia, the *ICAM1* gene transcription in the nuclei is increased and expression on the surface of endothelium cells is upregulated. ICAM-1 protein binding activity with leukocyte adhesion protein-1 (LFA-1) is increased and more lymphocytes from blood are transferred into cells of glomeruli and peritubular capillaries of the nephron in the kidney. Clinically, serum/plasma ICAM-1 levels are found to be increased and the proteins are released to urine, while injury in kidney glomeruli and tubular has occurred. Therefore, we suggest that heterozygosity of the *ICAM1* E469K polymorphism may confer an increased risk susceptibility in DKD.

Although the additional information concerning the genetic and biological effects of ICAM-1 in DKD in this article has been provided, there are still several questions remained. For instance, what are “the ICAM-1 protein molecular structure and biological function per see” in serum/plasma of DKD subjects with the ICAM1 E469K heterozygous genotype? How does ICAM-1 heterozygous protein bind with LFA-1 with higher activity and subsequently induces more lymphocytes transferred from blood into the cells of glomeruli and peritubular capillaries of the nephron in the kidneys? Further investigation to address these questions has been taken into our consideration.
